# Factors Contributing to Long-Term Severe Visual Impairment in Stevens-Johnson Syndrome and Toxic Epidermal Necrolysis

**DOI:** 10.1155/2017/2087578

**Published:** 2017-03-26

**Authors:** Passara Jongkhajornpong, Kaevalin Lekhanont, Sukanya Siriyotha, Silada Kanokrungsee, Varintorn Chuckpaiwong

**Affiliations:** ^1^Department of Ophthalmology, Ramathibodi Hospital, Mahidol University, Bangkok, Thailand; ^2^Department of Clinical Epidemiology and Biostatistics, Ramathibodi Hospital, Mahidol University, Bangkok, Thailand; ^3^Department of Dermatology, Ramathibodi Hospital, Mahidol University, Bangkok, Thailand

## Abstract

*Purpose*. To study the correlation between demographics and clinical variables and long-term severe visual impairment in patients with Stevens-Johnson syndrome (SJS) or toxic epidermal necrolysis (TEN). *Methods*. A retrospective chart review of SJS/TEN patients between 2004 and 2014 was conducted. Demographics, causative agents, ocular manifestations, and visual outcomes were collected. The data were analyzed using a multivariate logistic regression model. *Results*. Of the 89 patients including SJS (65, 73.03%), TEN (15, 16.85%), and SJS-TEN overlap (9, 10.11%), 55 were female. The mean age was 41.58 ± 19.17 years. The most common identified agents were medications. Among these groups, antibiotics were the most prevalent (47.19%). Three patients (3.7%) had unknown etiology. Antibiotics and nonpharmaceutical triggers were significantly associated with long-term severe visual impairment (odds ratio 4.32; *P* = 0.015 and 7.20; *P* = 0.037, resp.). There was a significant negative relationship between HIV infection and long-term severe visual impairment (*P* = 0.021). Among all chronic ocular complications, only corneal neovascularization significantly correlated with severe visual impairment (*P* = 0.001). *Conclusions*. SJS/TEN patients caused by nonpharmaceutical triggers or antibiotics have an increased risk of developing long-term severe visual impairment from corneal neovascularization. HIV infection might be a protective factor against long-term poor visual outcomes.

## 1. Introduction

Stevens-Johnson syndrome (SJS), toxic epidermal necrolysis (TEN), and SJS-TEN overlap are considered a spectrum of severe immunologic dermatobullous conditions involving the skin and mucosal membranes and usually triggered by drugs or infections [1, 2]. Although rare, these diseases are very important because they have high mortality and morbidity rates with ocular involvement often being the most serious long-term sequala [2, 3]. Ocular manifestations of SJS/TEN can be classified according to the clinical stages: acute and chronic. Initial eye involvement is highly variable and can range from self-limited conjunctival hyperemia to extensive sloughing of the entire ocular surface epithelium, including the tarsal conjunctiva and eyelid margin, leading to symblepharon formation, foreshortening of the fornix, and corneal ulceration or perforation [5]. Acute ocular complications developed in 50–81% of patients hospitalized for SJS/TEN [4–6]. Late ocular features include cicatricial changes of the conjunctiva and eyelids, severe dry eye, and ocular surface failure. These chronic complications are multifactorial in origin. They may not solely result from severe ocular inflammation during the acute phase but may also be results of a vicious cycle of microscopic injury due to severe dry eye and eyelid pathologies which are the permanent damage after the acute stage and recurrent episodes of conjunctival inflammation unrelated to mechanical irritation which may occur at some period following the acute disease episode [7, 8]. Chronic ocular sequelae occurred in up to 35% of patients with blinding corneal damage representing the most severe long-term complication for survivors of SJS/TEN [9]. Not all patients with early involvement end up with significant loss of vision. Although ocular sequelae are related to ocular involvement severity in the acute phase [6], chronic ocular complications after SJS/TEN can present with a variable course that is not always the direct consequence of initial inflammatory process [7]. Also, some patients without acute ocular involvement developed late complications [10]. These findings might reflect that there may be other external or internal factors associated with long-term ocular surface failure and severe visual impairment besides an acute ocular involvement score. Female gender and the acute systemic involvement score have been found to be prognostic factors predicting chronic ocular complications and the final best-corrected visual acuity (BCVA) [11]. Additionally, few studies have investigated the effect of the causative agents on chronic ocular damage [11–13]. However, the relationship of the causative drugs and chronic ocular complications is still controversial. Therefore, the purpose of this study is to assess the correlation between patients' demographics and clinical characteristics including the inciting agents and the severity of long-term visual impairment from SJS-/TEN-related chronic ocular complications in Thai population.

## 2. Methods

A single-center, retrospective chart review was conducted at Ramathibodi Hospital, Bangkok, Thailand, between December 2004 and December 2014. This study was approved by the Ethics Committee of Mahidol University School of Medicine and carried out in accordance with the Declaration of Helsinki. Informed consents were obtained from all subjects prior to the study.

Patients with SJS or TEN or SJS-TEN overlap were identified from hospital's electronic patient medical records and from the Cornea and Refractive Surgery Service database. The eligible criteria were patients with a dermatological diagnosis of SJS or TEN or SJS-TEN overlap and a follow-up time of at least 1 year. The classification criteria of Bastuji-Garin et al. were used to describe SJS, TEN, and overlap syndrome [14]. SJS was defined as detachment below 10% of the body surface area (BSA) plus widespread erythematous or purpuric macules or flat atypical targets; overlap SJS-TEN, detachment between 10% and 30% of the BSA plus widespread purpuric macules or flat atypical targets; TEN with spots, detachment above 30% of the BSA plus widespread purpuric macules or flat atypical targets; and TEN without spots, detachment above 10% of the BSA with large epidermal sheets and without any purpuric macule or target. Patients who had a history of other ocular surface diseases or ocular surgeries were excluded from the study.

We retrospectively reviewed the medical records and collected all demographics and clinical data of each patient including the information about the pathogenesis of the SJS or TEN and the potential inciting agents. The causative drugs were identified by a dermatologist and a pharmacist based on the detailed medical history and drug information gathered for 4 weeks before the disease onset and risk estimates for each drug adapted from Roujeau et al. [15]. The main outcome measures were best spectacle-corrected visual acuity (BSCVA) and chronic ocular complications at the final follow-up. The final BSCVA was obtained prior to corneal or limbal transplantation. Adapted from the World Health Organization (WHO) [16], in this study, severe visual impairment was defined as BSCVA of less than 20/200 with corresponding chronic ocular complications. Chronic ocular surface complications were broadly categorized into 3 groups: corneal (superficial punctate keratopathy, epithelial defect, loss of the palisades of Vogt, conjunctivalization, neovascularization, opacification, and keratinization); conjunctival (hyperemia, symblepharon formation); and eyelid (trichiasis, mucocutaneous junction involvement, meibomian gland involvement, and punctal damage) complications, according to the grading system for the evaluation of chronic ocular manifestations in patients with SJS established by Sotozono et al. [17]. The worse eye of each patient was chosen for statistical analysis.

Statistical analyses were performed with the statistical software package STATA version 14.1 (StataCorp, College Station, Texas, USA). Mean and standard deviation (SD) or median and range were used to describe continuous data. Frequency and percentage were used to describe categorical data. The chi-square test (or Fisher exact test) and *t*-test (or Mann-Whitney test) were used to compare patient demographics and the effects of causative agents on long-term visual outcomes. Univariate and multivariate logistic regression analyses were used to evaluate prognostic factors related to long-term severe visual impairment (BSCVA < 20/200). Multivariate logistic regression analysis was performed with variables whose *P* value was less than 0.1 in univariate analysis. The relationship between chronic ocular complications and severity of visual impairment was analyzed by the same methodology. Odds ratio (OR) with 95% confidence interval (CI) was reported. Statistical significance was considered at *P* < 0.05.

## 3. Results

Over a 10-year period, 89 patients with SJS/TEN were recruited into the study. There were 55 (61.80%) females and 34 (38.20%) males. The mean age at the first visit was 41.58 ± 19.17 years (range, 1 to 79 years). Sixty-five patients (73.03%) were classified as SJS, 15 patients (16.85%) as TEN, and 9 patients (10.11%) as SJS-TEN overlap. Thirty-three patients (43.82%) had been referred from local hospitals. Of the 89 patients, 67 patients (75.28%) presented with ocular involvement in the acute phase. Twenty-three patients (25.84%) recovered without significant long-term sequelae, and 44 patients (49.44%) developed chronic ocular complications. Severe visual impairment was found in 23 patients (25.84%). The median follow-up time was 6.4 years (range, 1 to 24.3 years). Fifty-one (57.30%) patients had underlying diseases, including hypertension (16 cases; 17.89%), human immunodeficiency virus (HIV) infection (13 cases; 19.7%), diabetes mellitus (DM) (11 cases; 12.36%), collagen vascular diseases (10 cases; 11.23%), dyslipidemia (5 cases; 5.62%), chronic kidney disease (5 cases; 5.62%), and liver disease (2 cases; 2.25%). Of the 10 patients with collagen vascular diseases, systemic lupus erythematous (SLE) was found in 7 cases (7.87%), mixed connective tissue disease (MCTD) in 2 cases (2.25%), and rheumatoid arthritis (RA) in 1 case (1.12%). Twelve out of 13 patients with HIV infection (92.86%) had a CD4 count less than 200 cells/*μ*l (mean, 120.54 ± 130.61; range, 5–496).

The inciting agents responsible for SJS/TEN are summarized in Table 1. Drugs were the leading cause of SJS/TEN (83 patients, 93.26%), followed by infection (3 patients, 3.37%), and unknown etiology (3 patients, 3.37%). There were 2 patients infected by herpes simplex virus and 1 patient infected by *Mycoplasma pneumoniae*. They presented with classic clinical signs and symptoms of infection with positive seroconversion without previous history of taking medications. Infection and unknown etiology in patients were grouped together as nonpharmaceutical triggers. Among all offending medications, antibiotics were the most common cause (47.19%), followed by anticonvulsants (25.84%), allopurinol (12.36%), and nonsteroidal anti-inflammatory drugs (NSAIDs) (10.11%). The frequent inciting antibiotics included penicillins (21.35%), sulfonamides (12.36%), tetracyclines (3.37%), and macrolides (3.37%). One offending medication was observed in 58 patients (64.04%), 2 possible offending medications in 22 patients (24.72%), and 3 possible offending medications in 3 patients (3.37%).

From the univariate analyses, there were no statistically significant associations between demographic variables such as sex, age, diagnosis, underlying diseases, and long-term visual outcomes except for the causative agents and HIV infection (Table 2). Regarding the causative agents, nonpharmaceutical triggers (infection and unknown etiology) and antibiotics were significantly correlated with long-term severe visual impairment with OR of 7.20 (95% CI, 1.13–45.96; *P* = 0.037) and 4.32 (95% CI, 1.44–14.02; *P* = 0.015), respectively. HIV infection was negatively correlated with long-term severe visual impairment (OR, 0.16; 95% CI, 0.03–0.71; *P* = 0.021). However, multivariate analysis could not be performed because no long-term severe visual impairment occurred in patients with HIV infection.

Of the 44 patients with chronic ocular complications, 23 patients (52.27%) ended up with severe visual impairment. Three major ocular surface complications including corneal, conjunctival, and eyelid complications as well as lens status are demonstrated in Table 3. The most common chronic ocular complication was superficial punctate keratopathy (79.55%), followed by corneal opacification (72.73%), and corneal neovascularization (56.82%). Univariate analysis showed that corneal neovascularization, symblepharon, and corneal opacification were significantly related to severe visual impairment (*P* < 0.05). From the multivariate model, only corneal neovascularization was significantly associated with severe visual impairment (OR, 15.66; 95% CI, 3.06–80.22; *P* = 0.001).

## 4. Discussion

It is well known that ocular involvement is very prevalent in SJS/TEN and can be severe and blinding. Nonetheless, because of the rarity of the disease, the small number of patients treated at any single center each year, the high mortality rate, and the rise of concern about acute ocular therapy to prevent late ocular complications, there have been limited studies focusing on the long-term ophthalmological sequelae of surviving patients [6–11, 18]. Although considerable advances have been made in recent years in the management of ocular manifestations of SJS/TEN in both acute and chronic stages of the disorder, some existing reports are contradictory, and delayed or inadequate treatments can result in irreversible ocular damage and loss of visual function [3]. Therefore, identification of predictive factors for subsequent ocular complications is necessary to improve both initial and further managements.

Our study demonstrated that in SJS/TEN patients, antibiotics were the most frequent inciting drug consistent with those seen in previous reports from other Asian countries [17, 18]. Causative agents and the underlying HIV infection were found to be the risk factors for severe visual impairment in the aftermath of the disease. The SJS/TEN patients induced by antibiotics alone and nonpharmaceutical triggers, including mycoplasma and herpes simplex infections or unidentified etiology, had 4.32 and 7.20 times greater risk of long-term poor visual outcomes, respectively. Meanwhile, anticonvulsants, allopurinol, NSAIDs, and other drugs did not show any statistically significant relationship to long-term severe visual impairment. These findings are similar to prior studies from Japan and Korea which reported that cephalosporin-related SJS/TEN exhibited relatively higher tendencies of experiencing severe acute ocular surface involvements, although not significant (0.05 < *P* < 0.07), and patients with SJS/TEN that were associated with other drugs such as carbamazepine, allopurinol, and quinolones were not likely to develop severe ocular surface involvements [19, 20]. However, NSAIDs have previously been observed to be potentially associated with chronic complications in a specific genetic background [12, 13]. In addition, a recent study found that SJS/TEN patients taking acetaminophen showed a significantly higher rate of experiencing severe ocular surface involvements than those taking other common causative drugs such as carbamazepine, allopurinol, and quinolones and the patients taking antipyretic analgesics, including acetaminophen and/or NSAIDs, for the treatment of common cold showed a higher frequency of experiencing severe ocular surface involvements compared with those taking antipyretic analgesics for the treatment of other diseases. Those results suggest that not only antipyretic analgesics but also viral infections causing cold-like symptoms may play some important roles in the development of severe ocular surface involvements [19]. Although that study looked into the relation of causative drugs to acute but not chronic ocular involvement, their observations give us a crucial idea that perhaps antipyretic analgesics which might have been prescribed along with antibiotics may actually influence the development of chronic ocular complications as well. Nevertheless, in this study, the information about acetaminophen use was not mentioned in any medical records and the prevalence of NSAID use was only 10.11%. This might be due to differences in self-medication patterns between various populations or a lack of awareness about the possibility of antipyretic analgesics being a precipitating factor of SJS/TEN so that clinicians did not inquire whether these drugs were administered before the onset of SJS/TEN. Also, cold medicine-related SJS/TEN patients with severe ocular complications have been found to be associated with certain types of HLA (*HLA-A*^∗^*02*:*06* and *HLA-A*^∗^*44*:*03*) and/or *IKZF1* [21]. Thus, the variation in genetic backgrounds, drug use behavior, and criteria for the identification of inciting agents between different studies could be the reasons for the diverse outcomes. Furthermore, as nonpharmaceutical triggers appeared to be correlated with long-term severe visual impairment, it should be emphasized that overlooked medications especially antipyretic analgesics, subclinical or unrecognized infections, or other unidentified external factors could be potential etiologies of SJS/TEN causing severe ocular involvements.

TEN and SJS are more common in acquired immunodeficiency syndrome (AIDS) patients. Consequent dry eye may also be further exacerbated by HIV-related lacrimal gland dysfunction [22]. However, it is interesting that none of our patients who had HIV infection developed long-term severe visual impairment. There was only one study in the literature investigating the effect of HIV infection on ocular involvements in SJS/TEN patients [23]. In that study, no statistically significant difference in the severity of chronic ocular complications was found between HIV-positive and HIV-negative patients [23]. In additional analysis in a subgroup of HIV-infected patients, there was no significant difference in severity scores by CD4 category [23]. Nevertheless, the study was conducted in a predominantly HIV-infected population (HIV-positive is 59.3% compared to 19.7% in our study), and intriguingly, most of their patients had low ocular severity scores and good Snellen visual acuities ranging from 20/20 to 20/60. Their results actually correspond to our finding that HIV infection might be a protective factor against poor visual outcomes in the long run. We hypothesized that the state of immunosuppression by blunting antigen-specific response in HIV-infected patients might be a key factor in preventing hyperresponsive innate immune storm that leads to severe ocular involvements in SJS/TEN patients. Moreover, 92.86% of our HIV-infected patients had a CD4 count less than 200 cells/*μ*l, and the only 1 patient with a CD4 count above 200 cells/*μ*l (496 cells/*μ*l) was being in the state of a rapid decline in CD4 level. These results support our hypothesis of immunosuppressive conditions potentially resulting in less severe ocular complications and favorable visual outcomes in HIV-infected patients. However, few case reports illustrated severe acute ocular complications from SJS in AIDS patients [24, 25]. In those 2 cases, the SJS-induced epithelial defects were complicated by superimposed corneal infections and eventually healed with a visual acuity of 20/60 in 1 case and blindness secondary to endophthalmitis in another case. Hence, opportunistic infections in patients with HIV disease might modify the SJS-/TEN-related ocular manifestations or make them worse, leading to severe visual loss.

Although approximately 50% of our patients developed chronic ocular complications which were greater than that of the earliest report [9], variable degrees of many types of ocular sequelae have been increasingly seen in later studies [6–8]. Our prevalence could then be underestimated, probably owing to little knowledge of chronic ocular complications of SJS/TEN in the past and the retrospective design of the study. The proportion of patients with severe visual impairment in the chronic phase was comparable with that of previous reports [10, 17, 26]. Corneal neovascularization was significantly associated with severe visual impairment similar to the prior study [17]. Nonetheless, corneal opacification was not included into the multivariate analysis because all patients with severe visual impairment presented with corneal opacity.

The limitations of this study included inherent shortcomings in any retrospective study, relatively small sample size, heterogeneous patient populations, and variable follow-up time. In addition, this study enrolled pediatric patients, in which the prognosis and the response for the treatment are significantly different from the adult group [27]. However, there were a very small number of pediatric patients in the current study. Another major drawback of this study is that acute ocular features and treatments in the acute phase were not included in its analysis. Also, antiviral drugs which are the common inciting agents associated with SJS/TEN among HIV populations were not studied in more details. These are all caused by incomplete information in medical records which could result in some confounding factors among the causative agents and the underlying diseases. Future multicenter, large-scale, prospective studies with genetic analysis are needed to clarify the natural history of SJS/TEN and risk factors associated with long-term poor visual prognosis.

## 5. Conclusion

In conclusion, SJS/TEN patients caused by nonpharmaceutical triggers or antibiotics appear to have an increased risk of developing long-term severe visual impairment from corneal neovascularization. HIV infection might be a protective factor against long-term poor visual outcomes. These results shed light on the critical role of particular inciting agents, baseline immune status, and possible other undiscovered factors contributing to the long-term visual outcomes in SJS/TEN. Thorough medical history taking and meticulous ocular examination are strongly recommended for all SJS/TEN patients to identify vulnerable and high-risk patients to develop serious ocular sequelae. Prompt ophthalmologist consultation and closely monitoring by corneal specialists are essential in order to prevent long-term severe visual impairment.

## Figures and Tables

**Table 1:** Causative agents associated with SJS/TEN patients and relative frequencies.

Inciting agents	Number of patients (*N* = 89)	Percentage (%)
Medications	83	93.26
Single possible drug	58	64.04
Antibiotics	25	28.09
Penicillins	8	8.99
Sulfonamides	8	8.99
Tetracyclines	2	2.25
Quinolones	1	1.12
Macrolides	1	1.12
Other antibiotics	5	5.62
Anticonvulsants	10	11.24
Carbamazepine	6	6.74
Phenytoin	3	3.37
Phenobarbital	1	1.12
Allopurinol	11	12.36
NSAIDs	4	4.49
Antimalarials	2	2.25
Antivirals	2	2.25
Other drugs	4	4.49
Multiple possible drugs^∗^	25	28.09
Multiple antibiotics	7	7.87
Antibiotics + anticonvulsants	5	5.62
Antibiotics + NSAIDs	2	2.25
Antibiotics + antivirals	1	1.12
Antibiotics + bromhexine	1	1.12
Multiple nonantibiotics	9	10.11
Nonpharmaceutical triggers	6	6.74
Infections	3	3.37
Mycoplasma	1	1.12
Herpes simplex	2	2.25
Unidentified	3	3.37
Total	89	100

^∗^More than one possible drug was identified.

**Table 2:** Analysis of the correlation between demographics and severe visual impairment in SJS/TEN patients (*n* = 89).

Variables	Nonsevere visual impairment (%) (*n* = 66, 74.16%)	Severe visual impairment (%) (*n* = 23, 25.84%)	OR (95% CI)	*P* value
Sex				
Male Female	25 (37.88) 41 (62.12)	9 (39.13) 14 (60.87)	1.05 (0.40–2.79) 1.00	0.915
Age (years)				
<40 ≥40	28 (42.42) 38 (57.58)	13 (56.52) 10 (43.48)	1.76 (0.68–4.60) 1.00	0.245
Diagnosis				
Overlap/TEN SJS	19 (28.79) 47 (77.21)	5 (21.74) 18 (78.26)	0.68 (0.22–2.12) 1.00	0.512
Diabetes mellitus				
Yes No	9 (13.64) 57 (86.36)	2 (8.70) 21 (91.30)	0.60 (0.12–3.02) 1.00	0.539
Autoimmune diseases				
Yes No	7 (10.61) 59 (89.39)	3 (13.04) 20 (86.96)	1.26 (0.30–5.36) 1.00	0.750
HIV infection				
Yes No	13 (19.7) 53 (80.30)	0 (0) 23 (100)	0.16 (0.03–0.71) 1.00	0.021^∗^
Causative agents				
Nonpharmaceutical triggers	3 (4.55)	3 (13.04)	7.20 (1.13–45.96)	0.037^∗^
Antibiotics alone	20 (30.30)	12 (52.17)	4.32 (1.44–14.02)	0.015^∗^
Antibiotics with others	7 (10.61)	3 (13.04)	3.09 (0.59–15.98)	0.179
Nonantibiotics	36 (54.55)	5 (21.74)	1.00	

^∗^Statistical significance *P* < 0.05; HIV: human immunodeficiency virus.

**Table 3:** Analysis of the correlation between ocular findings and severe visual impairment in SJS/TEN patients with chronic ocular complications (*n* = 44).

Ocular findings	Nonsevere visual impairment (%) *n* (%), *n* = 21	Severe visual impairment (%) *n* (%), *n* = 23	Univariate analysis		Multivariate analysis	
			OR (95% CI)	*P* value	OR (95% CI)	*P* value
*Corneal complications*						
SPK						
Yes No	19 (90.48) 2 (9.52)	16 (69.57) 7 (30.43)	0.60 (0.12–3.02) 1.00	0.539		
Corneal opacification						
Yes No	9 (42.86) 12 (57.14)	23 (100) 0 (0)	2.56 (1.18–5.52) 1.00	0.017^∗^		
Corneal neovascularization						
Yes No	5 (23.81) 16 (76.19)	20 (86.96) 3 (13.04)	21.33 (4.42–103.06) 1.00	<0.001^∗∗^	15.66 (3.06–80.22)	0.001^∗^
*Conjunctival complications*						
Hyperemia						
Yes No	7 (33.33) 14 (66.67)	13 (56.52) 10 (43.48)	2.60 (0.76–8.86) 1.00	0.127		
Symblepharon						
Yes No	3 (14.29) 18 (85.71)	12 (52.17) 11 (47.83)	6.55 (1.50–28.49) 1.00	0.012^∗^	2.756 (0.48–15.92)	0.257
*Eyelid complications*						
Trichiasis						
Yes No	7 (33.33) 14 (66.67)	9 (39.13) 14 (60.87)	1.29 (0.37–4.42) 1.00	0.690		
MGD						
Yes No	8 (38.10) 13 (61.90)	6 (26.09) 17 (73.91)	1.74 (0.48–6.28) 1.00	0.395		
*Lens status*						
Cataract No cataract	4 (19.05) 17 (80.95)	4 (17.39) 19 (82.61)	0.89 (0.58–2.15) 1.00	0.887		

^∗^Statistical significance *P* < 0.05. ^∗∗^Statistical significance *P* < 0.01; MGD: meibomian gland dysfunction; SPK: superficial punctate keratopathy.
